# 

**Published:** 1918-11-23

**Authors:** 


### Influenza: A French Doctor's Experiment.

M. Roux, Director of the Pasteur Institute, is reported in the French press to have rendered an account to the Académie des Sciences of an experiment made upon himself by Doctor Dujarie de la Rivière. After having filtered blood from a patient attacked by influenza, this doctor injected some of the liquid under his skin. At the end of three or four days the symptoms of influenza appeared, but apparently he treated them successfully. Ten days afterwards, wishing to establish the fact of his constitutional immunity, he plastered his throat and nares with the expectorations of another influenza patient. Nothing is said to have come of it, and the experiment is quoted to support the theory that recovered sufferers from influenza are protected from a relapse through direct contagion.

### The Welfare of Infancy.

The third of a series of three lectures on the Feeding of Children will be given, under the auspices of the National Association for the Prevention of Infant Mortality and for the Welfare of Infancy, by Dr. Eric Pritchard at Dr. Williams' Library, Gordon Square, W.C., on November 26. Tickets may be obtained from Miss Halford, Secretary, National Association for the Prevention of Infant Mortality, 4 Tavistock Square, W.C. 1.

### Women on Governing Bodies.

It has been decided at a general meeting of the Institute of Hygiene to elect women to the governing bodies of the Institute.

### The College of Nursing

**(Limited by Guarantee).**

### EDINBURGH CENTRE.

(Joint Hon. Secs. Miss Turnbull, R.R.C., and Miss Cathcart, "The Elms," Whitehouse Loan.)

First Public Meeting.

The Edinburgh Centre of the College is to be congratulated on its first public meeting, which was held in the City Chambers, Edinburgh, at 3.30 p.m., on Tuesday, November 12. The general rejoicing over the dawn of peace did not deter a large and representative company of nurses from crowding the hall. Amongst them were matrons and sisters from the larger institutions, members of the Q.V.J.I.N., private nurses, nurses engaged in health visiting, and many nurses in training. Miss Gill, R.R.C., President of the Centre, introduced the speaker, Dr. Mary Macnicol, and explained that the National Council for Combating Venereal Diseases, which was holding a six weeks' campaign in the city, had approached her as to how the interest of nurses could be enlisted. She had been glad to feel that there was a representative organisation ready to arrange for meetings, and suggested that the Edinburgh Centre should invite the nurses if the National Council would provide speakers for a series of six lectures.

Dr. Macnicol said that in dealing with her subject, "The Responsibility of Citizenship," she would only touch on those aspects of it which were connected with venereal diseases. She showed how widespread such diseases were, and justified the position of the Royal Commission, held just before the war, which urged that the public should be educated in these matters, and that the conspiracy of silence should be broken down. She clearly demonstrated the sinister effect of syphilis and gonorrhœa on the life, health, and sanity of the nation, and emphatically asserted that both of these diseases were curable and must be stamped out. Treatment to be successful, she said, must be early, persistent, and skilful, and she urged upon nurses the necessity of showing the utmost kindness and consideration toward those suffering from venereal diseases, as, if we did not make the clinics more attractive than heretofore, only patients in the advanced stages would come to be treated. Now that women were enfranchised they must see that the horrible waste caused by such diseases was made to cease, and they must not relax their efforts till the great fight against this deadly foe was fought and won.

In a few well-chosen words Miss Sandford, a former matron of the Edinburgh City Hospital, thanked Dr. Mary Macnicol for her most interesting and inspiring address.

Forthcoming Lectures.

The following lectures have been arranged under the auspices of the National Council for Combating Venereal Diseases:—

November 19, "The Renewal of Life," Dr. Marion Newbigin, D.Sc. (lantern illustrations); November 26, "Venereal Diseases," Dr. Mary Macnicol; December 3, "Medical and Legal Prevention," Dr. Mary Douie; December 10, "Social Prevention," Mrs. Altentop; December 17, "Marriage and Parenthood," Mrs. Chalmers Watson, C.B.E., M.D.

All the lectures are to be held in the City Chambers at 3.30 p.m., and all nurses, whether members of the Centre or not, are welcome.

### BIRMINGHAM CENTRE.

(Hon. Secretary, Miss M. I. Colburn, 94 Moseley Road.)

Tuesday, December 10, 6 p.m., at the Birmingham General Hospital.—Lecture by Captain Wynn on "The Modern Treatment of Pneumonia." All nurses are cordially welcomed, non-members of the College being charged 1s.

On Friday, November 15, Miss Cowlin, Organising Secretary of the College, had a large and interested audience at the General Hospital, Birmingham, for her address on "The Aims and Objects of the College of Nursing," which was much appreciated by those present.

### CAMBRIDGE CENTRE.

(Hon. Secretary, Miss K. Newman, Addenbrooke's Hospital.)

The following lectures have been arranged:—

November 29, 5.30 p.m., at the 1st Eastern General Hospital Recreation Room.—Lecture, with lantern slides, by Miss E. R. Saunders, of Newnham College, "Our Knowledge of Heredity Before and After 1900."

December 2, 3 p.m., at Addenbrooke's Hospital.—General meeting for members of the nursing profession to discuss the objects and work of the local Centre and other matters of interest. All members of the local Centre are asked to attend and bring a non-member with them.

January 2, 1919, 3 p.m., at the 1st Eastern General Hospital Recreation Room.—Lecture, with lantern slides, by Professor Seward, Master of Downing College, "Plant Life."

January 30, at the 1st Eastern General Hospital.—Lecture, with lantern slides, by Professor Nuttall, F.R.S.

Nurses not members of the local Centre are invited to the lectures on payment of 6d. for each lecture.

### Meetings in the North.

### INFLUENTIAL MEETING AT YORK.

An influential meeting was held at St. William's College Hall, York, on Thursday, November 14, when an address on the work of the College was given by Miss Cowlin. The chair was taken by Mr. W. F. H. Thomson, J.P., Chairman of the County Hospital, York, and accompanying him were the Very Rev. Dean of York (Dr. Foxley Norris); Mr. Charles Lupton, Chairman and Treasurer of the Leeds General Infirmary; and Mr. F. Neden, Secretary of the County Hospital, York.

The Chairman said he supposed that at the present time there was no civilian who did not acknowledge that he owed a tremendous debt of gratitude to the nurses of this country. Their work both at home and abroad was magnificent and heroic, and they were deserving of our most grateful thanks.

Miss Cowlin emphasised the fact that the College stood for State Registration as a protection both for the public and the trained nurse, and it stood also for the higher education of nurses. The College realised that women did not enter the nursing profession in order to obtain halos, but because it was the finest profession open to women, and to render fine service they needed to work under fair conditions, and were deserving of just recognition. The Council of the College was in the hands of the nurses on the Register, and it should be possible, through the Local Centres of the College, to make this Council representative of every branch of Nursing.

On the motion of Mr. Charles Lupton, the following resolution was passed:—

> That this meeting, having heard the objects of the College of Nursing, Limited, cordially approve the scheme, which will tend to raise the status of nurses generally by the adoption of State Registration, thus securing Government recognition, and also by the establishment of a one-portal examination which will ensure a uniform standard of efficiency throughout the members of the profession.

Meetings at Newcastle and Sunderland.

Miss Cowlin addressed meetings at the Royal Victoria Infirmary, Newcastle, on November 12, and at the Royal Infirmary, Sunderland, on November 13. In answer to questions, Miss Cowlin said that apart from the Hon. Sir Arthur Stanley, who was Chairman of the College, the signatories to the Articles of Association were not members of the Council, which was an elective Council, and by 1920 would have been placed there by the nurses on the Register.

